# Population Genetics of *Nosema apis* and *Nosema ceranae*: One Host (*Apis mellifera*) and Two Different Histories

**DOI:** 10.1371/journal.pone.0145609

**Published:** 2015-12-31

**Authors:** Xulio Maside, Tamara Gómez-Moracho, Laura Jara, Raquel Martín-Hernández, Pilar De la Rúa, Mariano Higes, Carolina Bartolomé

**Affiliations:** 1 Medicina Xenómica, CIMUS, Universidade de Santiago de Compostela, Santiago de Compostela, Galicia, Spain; 2 Xenómica Comparada de Parásitos Humanos, IDIS, Santiago de Compostela, Galicia, Spain; 3 Departamento de Anatomía Patolóxica e Ciencias Forenses, Universidade de Santiago de Compostela, Santiago de Compostela, Galicia, Spain; 4 Laboratorio de Patología Apícola. Centro de Investigación Apícola y Agroambiental (CIAPA), Instituto Regional de Investigación y Desarrollo Agroalimentario y Forestal (IRIAF), Consejería de Agricultura de la Junta de Comunidades de Castilla-La Mancha, Marchamalo, Guadalajara, Spain; 5 Departamento de Zoología y Antropología Física, Facultad de Veterinaria, Universidad de Murcia, Murcia, Spain; 6 Instituto de Recursos Humanos para la Ciencia y la Tecnología (INCRECYT-FEDER), Fundación Parque Científico y Tecnológico de Albacete, Albacete, Spain; University of Ottawa, CANADA

## Abstract

Two microsporidians are known to infect honey bees: *Nosema apis* and *Nosema ceranae*. Whereas population genetics data for the latter have been released in the last few years, such information is still missing for *N*. *apis*. Here we analyze the patterns of nucleotide polymorphism at three single-copy loci (*PTP2*, *PTP3* and *RPB1*) in a collection of *Apis mellifera* isolates from all over the world, naturally infected either with *N*. *apis* (*N* = 22) or *N*. *ceranae* (*N* = 23), to provide new insights into the genetic diversity, demography and evolution of *N*. *apis*, as well as to compare them with evidence from *N*. *ceranae*. Neutral variation in *N*. *apis* and *N*. *ceranae* is of the order of 1%. This amount of diversity suggests that there is no substantial differentiation between the genetic content of the two nuclei present in these parasites, and evidence for genetic recombination provides a putative mechanism for the flow of genetic information between chromosomes. The analysis of the frequency spectrum of neutral variants reveals a significant surplus of low frequency variants, particularly in *N*. *ceranae*, and suggests that the populations of the two pathogens are not in mutation-drift equilibrium and that they have experienced a population expansion. Most of the variation in both species occurs within honey bee colonies (between 62%-90% of the total genetic variance), although in *N*. *apis* there is evidence for differentiation between parasites isolated from distinct *A*. *mellifera* lineages (20%-34% of the total variance), specifically between those collected from lineages A and C (or M). This scenario is consistent with a long-term host-parasite relationship and contrasts with the lack of differentiation observed among host-lineages in *N*. *ceranae* (< 4% of the variance), which suggests that the spread of this emergent pathogen throughout the *A*. *mellifera* worldwide population is a recent event.

## Introduction

The genus *Nosema* (Fungi, Microsporidia, Dihaplophasea, Dissociodihaplophasida Nosematidae; Nägeli, 1857) contains over eighty species [1,2] typically found in arthropods. Two species, *N*. *apis* and *N*. *ceranae*, parasitize the Western honey bee, *Apis mellifera*. *N*. *apis* Zander, 1909 is a globally distributed pathogen that was identified in this host more than a hundred years ago [3], whereas *N*. *ceranae* was described at the end of the twentieth century [4]. This latter species, although initially discovered in the Asian honey bee *Apis cerana* [4], was recently proved to infect *A*. *mellifera* [5,6], and since then it has been found worldwide in this new host [7,8,9,10,11], as well as in several other *Apis* [12,13] and *Bombus* species [14,15]. Both pathogens are transmitted through the ingestion of spores during feeding, grooming and trophallaxis [16,17]. Once in the gut, they invade the ventricular cells causing disease, but the clinical and epidemiological characteristics of the parasitization by either species are different; the infection by *N*. *apis* (type A nosemosis) does not usually cause the death of the colonies and is characterized by dysentery, general weakening of the adults, locomotion impairment and crawling [18]. These symptoms are not present in *N*. *ceranae* infections (type C nosemosis) [19], which produce alterations in the temporal polyethism, foraging activity and life span of infected bees [20,21,22]. Although the same could also be true for *N*. *apis* [23], the higher prevalence of *N*. *ceranae* throughout the year in temperate climates [24,25]–in contrast with that of *N*. *apis*, which usually displays seasonal peaks [26]–, induces a chronic stress on the colony that may eventually lead to its collapse [20,27,28]. This effect, whose potential relationship with the large scale depopulation phenomenon is still matter of debate ([24,28], or [29,30] for a different point of view), is much more dramatic in Mediterranean countries, especially in Spain, where climatic conditions and/or beekeeping practices seem to increase the impact of *N*. *ceranae* on honey bee colonies reviewed in [31].

Genetic data revealed that *N*. *apis* and *N*. *ceranae* are highly divergent at the nucleotide level (average nonsynonymous divergence of 10%; [32]) and that there has been considerable gene shuffling since the split from their common ancestor [33], evidencing that they are not very close relatives within the genus *Nosema* [34,35].

The genetic characterization of *N*. *ceranae* populations in *A*. *mellifera* has been achieved in the last few years by analyzing different components of the ribosomal DNA (rDNA) [36,37], single-copy genes [32,38,39,40,41] and whole genomes [42]. The most relevant conclusions of these studies are that i) *N*. *ceranae* isolates obtained from individual honey bees exhibit multiple alleles at single copy loci [38,39], ii) most of the variation resides within honey bee colonies [39,42], iii) there is no differentiation among geographically distant isolates [36,38,39,42], iv) this pathogen has experienced a recent demographic expansion in *A*. *mellifera* [38,39,42], and v) there is evidence for low, but significant, levels of recombination [36,38,40,41,42].

In contrast, there is little information about the population genetics of *N*. *apis*. Although a few sequence data have been released in public databases, most of them remain unpublished (e.g. PopSets 723438493, 698364701, 225055863 from GenBank) and/or involve the analysis of rDNA [43,44,45] that harbors multiple copies in the *N*. *apis* genome [33]. These are organized as tandemly repeated units, each of them consisting of a small (SSU) and large (LSU) subunits separated by an internal transcribed spacer (ITS), and an intergenic spacer (IGS) [45,46] (see [33] for a slightly different organization). The redundancy of these arrays usually promotes the conservation of rDNA sequences through different mechanisms (concerted evolution [47], and/or birth-and-death processes [44]) that preserve their important role in mRNA translation. However, in the case of *N*. *apis* and *N*. *ceranae*, rDNA copies are highly diverse [33,36,48] and, although the reasons for the existence of differently expressed rRNA copies are still to be determined [37], the presence of such heterogeneous units complicates the assessment of the levels of polymorphism [44,49], as within-genome heterogeneity is hard to disentangle from between-individuals diversity. This limits the use of ribosomal loci to estimate the genome-wide patterns of variability.

Here we report a population genetic analysis conducted to address questions that are central to our understanding of the recent evolutionary history of *N*. *apis*, such as: what are the levels of genetic variation of this parasite? Is there any genetic evidence for a long-term association between *N*. *apis* and *A*. *mellifera*? Is the *N*. *apis* population panmictic or is there any sign of geographical structure? With this aim, the sequences of three single copy genes were studied in a collection of *N*. *apis* and *N*. *ceranae* isolates obtained from *A*. *mellifera* colonies from all over the world. These loci had been previously studied in *N*. *ceranae* [32,39,40] and their patterns of polymorphism used to yield new insights into this parasite’s populations. Our results, along with these previous data, provide the first comparative analysis of the patterns of genetic variation of both pathogens in the same host species.

## Material and Methods

### Samples

*N*. *apis* was isolated from twenty two naturally infected *A*. *mellifera* colonies from eleven countries worldwide: Algeria, Argentina, Canada, Chile, Germany, Hungary, the Netherlands, Poland, Slovenia, Spain, and Turkey (S1 Table). A similar number of *N*. *ceranae* isolates (N = 23) were collected from *A*. *mellifera* colonies from 17 countries: Algeria, Argentina, Australia, Brazil, Canada, Chile, Croatia, Greece, Hawaii (USA), Hungary, Japan, the Netherlands, Slovenia, Solomon Islands, Spain, Taiwan, and continental United States of America (S2 Table).

### Ethics statement

No specific permits were required for the described studies, which did not involve endangered or protected species.

### DNA extraction, PCR amplification, cloning and sequencing

DNA was extracted from homogenized pools of 15–20 honey bees from each colony. This was carried out as in [50], using the BioSprintTM 96 DNA Blood Kit (QIAgen, Izasa, Barcelona, Spain). The reagents used in this process were tested by PCR to check for the presence of potential contamination with *N*. *apis*, *N*. *ceranae* or honey bee DNA in each round of extractions. The identity of *Nosema* species was determined by specific PCR amplification of the *16S rDNA*, as in [51]. No co-infections were detected in these samples.

Specific primers were designed with Primer Blast (http://www.ncbi.nlm.nih.gov/tools/primer-blast/index.cgi?LINK_LOC=BlastHome) using sequences of each species as query (KE647054.1: 13328–14116—locus tag NAPIS_ORF00435—for *PTP2*, KE647278.1: 2294–4285—locus tag NAPIS_ORF01922—for *PTP3* and DQ996230.1 for *RPB1* in *N*. *apis*, and XM_002995356.1 for *RPB1* in *N*. *ceranae*, respectively). Primer pairs for amplification of *PTP2*, *PTP3* and *RPB1* in *N*. *apis* were: PTP2 Na-F (CTGCTACAGCACCGCCATTA) and PTP2 Na-R (TGGGGTTTAATCTTGCTTTTTCCA), PTP3 Na-F (AGACAAGGTGTTTCTCCAAAAGA) and PTP3 Na-R (GCAAGGTTTTCTTCTGTTGCC) and RPB1 Na-F (GTTAAGAGCAGAAGATGATCTAAC) and RPB1 Na-R (CTGATAATTTGTTTTCCTGTCCAATA), respectively. Primer pairs to amplify *RPB1* in *N*. *ceranae* were those published in [32].

PCR amplification, cloning and sequencing procedures were performed as in [39]. PCR annealing temperatures were adjusted for each of the primer pairs. These were 59.0°C, 58.0°C and 56.5°C for *PTP2*, *PTP3* and *RPB1* in *N*. *apis* and 55°C for *RPB1* in *N*. *ceranae*, respectively. Each round of PCR amplification included negative and positive controls (PCR components with no template DNA, and PCR components + DNA extracted from *N*. *ceranae*–or *N*. *apis*–positive isolates, respectively).

Sequences were checked for accurate base calling using CodonCode Aligner (CodonCode Corporation, Dedham, MA, USA) and aligned with MUSCLE [52] with their reference sequences to determine the nucleotide positions at each locus. The alignments were manually corrected with BioEdit [53] and the sequences submitted to GenBank (S1 and S2 Tables).

### *Apis mellifera*: lineage assignment

Determination of the *A*. *mellifera* evolutionary lineage was performed by sequence analysis of the intergenic region between the tRNA^leu^ and the cytochrome oxidase II (cox2) gene as described previously [54]. DNA was extracted from a pair of legs using the Chelex^®^ method [55]. The intergenic tRNA^leu^-cox2 region was PCR-amplified in a thermocycler PTC 100 (MJ Research) in a total volume of 12.5 μL with KapaTaq DNA Polymerase (KAPA BIOSYSTEMS), containing 2 μL of DNA template, 200 μM total dNTP, 1 X Reaction Buffer, 0.5 U/rxn KapaTaq DNA Polymerase, 1.5 mM MgCl_2_, 0.4 μM of each primer (E2 and H2, [56]). The thermocycler program used was: 94°C (5 min); 35 cycles of a 45 s denaturation at 94°C, a 45 s elongation at 48°C, a 60 s extension at 62°C; and a final extension step at 65°C for 20 min. Amplicons were sequenced with the primer E2 (Secugen S.L., Madrid, Spain). Each sequence was manually checked for base calling and a multiple sequence alignment was performed with the MEGA program, version 6 [57]. Evolutionary lineages were determined by comparison with sequences deposited in GenBank (lineage C: JQ977699, JF723946; lineage M: HQ337441, KF274627; lineage A: EF033650, JQ746693).

### Sequence analyses

Nucleotide diversity in *N*. *apis* and *N*. *ceranae* was estimated at synonymous and non-synonymous sites with DnaSP v5.10.02 [58]. Sites with alignment gaps were excluded from the analyses. *π* [59] and *θ*_W_ [60] were calculated applying the Jukes-Cantor correction [61]. *π*, the average number of pairwise differences between sequences, is sensitive to the frequency of polymorphisms and complementary to the estimate of *θ*_W_, which measures the levels of variability by counting the number of segregating sites, independently of their frequency, and thus giving more weight to rare variants. The Tajima´s *D* test [62] compares the two statistics. If the population is in mutation-drift equilibrium, *π* and *θ*_W_ are expected to have same value, and *D* should be equal to zero. Negative *D* values reflect an excess of low frequency variants (greater *θ*_W_), which under neutrality can be interpreted as evidence for a recent population expansion. According to Tajima’s considerations on the different distributions followed by *D* at individual or pooled loci [62], the statistical significance of the deviation from neutral expectations for individual genes was determined using DnaSP (which assumes a beta distribution). When several unlinked regions of DNA were combined to describe the patterns of polymorphism of a species (pooled loci) this significance was calculated by applying Tajima´s formulae and assuming a normal distribution [62]. The possibility of a population expansion was further investigated by applying the Fu’s *F*_*S*_ [63], as implemented in DnaSP v5.10.02. Its significance was assessed by comparing the observed values with a null distribution generated by 1,000 coalescent simulations. The number of net nucleotide substitutions per site between populations, *D*a [59], was also estimated with DnaSP.

The program MLHKA [64] allows to test for selection at individual loci in a multilocus framework by comparing the relative amounts of within and between-species synonymous variation across unlinked loci. [65]. The patterns of diversity at the genes used in this study were compared with those observed at other loci with similar data available (*actin*, *Hsp70*, *HSWP4*, and *SWP30*) [32,38,39]. Genes that did not exhibit enough sequence identity between *N*. *apis* and *N*. *ceranae* to be confidently assigned as orthologs were discarded from the analysis (*NCER_100064*, *NCER_100070*, *NCER_100533*, *NCER_100768*, *NCER_101165* and *NCER_101600*; [38]), as was *PTP1* [39], which is tightly linked to *PTP2* [48,66]. Rates of synonymous and nonsynonymous divergence between *N*. *apis* and *N*. *ceranae* sequences for these loci were estimated using the Yang and Nielsen method [67], as implemented in the software PAML v 4.8a [68].

Lower bounds of the recombination rate were estimated using two different statistics under the assumption of the infinite sites model (i.e. each segregating site has mutated only once): *Rm*, the minimum number of recombination events, is based on the four-gamete test [69], which infers a recombination event if all four possible two-locus haplotypes occur in the sample, and *Rh* [70], which bounds the number of recombination events by calculating the difference between the number of haplotypes in the sample and the number of segregating sites. Both statistics were calculated with RecMin [70] (http://www.stats.ox.ac.uk/~myers/RecMin.html). The population scaled recombination rate (*ρ*) at the three loci was estimated applying the composite-likelihood method of Hudson [71], adapted to finite-sites model (to account for sites that might have experienced more than one mutation), as implemented in LDhat v2.0 [72]. Since the likelihood of observing recombination is dependent on the order of sites, the statistical significance of a non-zero rate of recombination was evaluated with a permutation test, in which the maximum composite likelihood was calculated under random permutation of the physical position of the variants (1000 permutations) [72]. *Nosema* parasites are commonly found as single-cell diplokaryons, so that they harbor a minimum of two haploid genomes. Thus, the number of haploid genomes is assumed to be 2 x 2*N*_*e*_, and *ρ* = 4*N*_*e*_ *r*.

Haplotype diversity was estimated with DnaSP v5.10.02, the statistics *K*_ST_* [73] and *S*_nn_ [74], were used to investigate the population structure. Their significance was assessed using permutation tests (1000 replicates).

An analysis of molecular variance (AMOVA) was performed with Arlequin 3.5 [75] and the significance of covariance components was checked by applying non-parametric permutation procedures (3000 permutations).

Haplotype networks were generated with Network 4.6.1.0 (Fluxus Technology, http://www.fluxus-engineering.com/sharenet.htm) using the Median-Joining algorithm, which allows for more than one different nucleotide per site. The epsilon parameter was set to 0, 10 and 20 in successive runs in order for the resulting network to include all possible shortest trees in the resulting network. Since no significant differences were observed, only those networks generated with epsilon = 0 are presented. The Connection Host criterion was used as distance calculation method. The Reduced Median algorithm (with *r* = 2) was applied to obtain a simplified network containing all shortest trees. All networks were redrawn manually.

### Identification of mating and meiotic genes

The identification in *N*. *apis* and *N*. *ceranae* of the components of a sex-related locus [76] and an inventory of genes involved in meiosis [77] was performed by means of Blastp and SQR Sequence Search (https://www.ncbi.nlm.nih.gov/Structure/seqr) using as queries protein sequences from other microsporidia such as *Encephalitozoon cuniculi*, *Enterocytozoon bieneusi*, *Antonospora locustae* and *Nosema bombycis*.

## Results

### Genetic diversity

The genetic variability of *N*. *apis* samples was initially assessed at *PTP2*, *PTP3* and *RPB1* in seven naturally infected *A*. *mellifera* colonies from Algeria, Argentina, Canada, Slovenia, Spain (*N* = 2) and Turkey (dataset^A^ in Table 1 and S1 Table). To increase the resolution of the analysis, and given that the levels of diversity were similar for the three genes (see below), the *RPB1* locus was randomly selected to enlarge the former dataset with sequences of 15 additional samples from Canada (*N* = 2), Chile (*N* = 2), Germany (*N* = 3), Hungary, Netherlands (*N* = 2), Poland (*N* = 3), Slovenia (*N* = 2), (dataset^A + B^ in Table 1 and S1 Table).

**Table 1:** Nucleotide diversity at three loci from *N*. *apis*: *PTP2*, *PTP3* and *RPB1*.

			*PTP2*							*PTP3*							*RPB1*						
	Isolate	Origin	*N*	*π*_S_	*θ*_WS_	*D*_S_	*π*_A_	*θ*_WA_	*D*_A_	*N*	*π*_S_	*θ*_WS_	*D*_S_	*π*_A_	*θ*_WA_	*D*_A_	*N*	*π*_S_	*θ*_WS_	*D*_S_	*π*_A_	*θ*_WA_	*D*_A_
^A^	52	SLO	9	0.31	0.51	-1.36	0.00	0.00	NA	5	0.00	0.00	NA	0.00	0.00	NA	9	1.78	1.42	1.03	0.15	0.25	-1.68
	174	TUR	10	0.28	0.49	-1.40	0.15	0.26	-1.67	5	1.05	1.25	-1.05	0.34	0.41	-1.09	10	0.71	1.02	-1.28	0.11	0.19	-1.67
	204	ARG	8	0.00	0.00	NA	0.09	0.14	-1.31	5	1.42	1.68	-1.09	0.13	0.10	-1.22	11	0.72	0.65	0.34	0.20	0.23	-0.49
	381	CAN	9	0.31	0.51	-1.36	0.12	0.20	-1.51	10	0.53	0.93	-1.56	0.24	0.30	-0.76	11	1.22	2.13	-1.90*	0.32	0.51	-1.59
	399	SPA	7	1.60	1.69	-0.34	0.16	0.23	-1.36	7	1.02	1.42	-1.43	0.12	0.09	1.34	7	0.14	0.20	-1.01	0.12	0.17	-1.36
	410	SPA	10	1.79	1.46	0.85	0.15	0.26	-1.67	13	1.22	0.84	1.37	0.13	0.20	-1.23	12	1.06	0.63	2.28*	0.07	0.14	-1.63
	854	ALG	10	1.30	0.98	1.23	0.04	0.07	-1.11	5	0.70	0.83	-0.97	0.17	0.20	-0.97	8	0.12	0.18	-1.05	0.03	0.05	-1.05
	Pooled ^A^		63	1.17	1.91	-1.53	0.10	0.58	-2.51***	50	1.27	2.14	-1.20	0.20	0.57	-1.91*	68	1.50	1.81	-0.55	0.25	0.74	-2.08*
^B^	59	SLO															8	1.97	1.30	2.29*	0.29	0.32	-0.34
	264	POL															10	1.94	1.36	1.78	0.18	0.14	0.85
	266	POL															10	1.96	1.70	0.58	0.21	0.29	-1.19
	268	POL															10	1.93	1.89	1.03	0.17	0.14	0.70
	363	CHI															8	2.09	1.66	1.10	0.18	0.16	0.58
	380	CAN															8	1.80	1.48	0.93	0.19	0.21	-0.52
	382	CAN															10	1.83	1.19	-2.16*	0.23	0.24	-0.23
	411	CHI															10	0.86	1.36	-1.64	0.16	0.29	-1.80*
	529	SLO															9	1.66	1.77	-0.37	0.09	0.15	-1.51
	569	HUN															10	1.93	1.36	1.69	0.20	0.19	0.14
	1074	GER															8	2.07	1.48	1.77	0.17	0.26	-1.60
	1098	GER															10	1.32	1.36	-0.21	0.18	0.24	-1.04
	1099	GER															10	0.79	0.85	-0.33	0.15	0.19	-0.94
	1511	NED															10	2.03	1.53	1.31	0.10	0.10	0.02
	1735	NED															9	0.11	0.18	-1.09	0.06	0.10	-1.36
	Pooled ^A^ ^+^ ^B^																208	1.68	2.78	-1.16	0.22	1.37	-2.53***

^A^, Seven isolates used to estimate *N*. *apis* diversity;

^B^, 15 additional isolates used for further analysis of *RPB1*;

ALG: Algeria, ARG: Argentina, CAN: Canada, CHI: Chile, GER: Germany, HUN: Hungary, NED: Netherlands, POL: Poland, SLO: Slovenia, SPA: Spain, TUR: Turkey; *N*: number of cloned sequences; *π*_S_ and *π*_A_, pairwise nucleotide diversity at synonymous and nonsynonymous sites expressed as percentage, respectively [59]; *θ*_WS_ and *θ*_WA_, nucleotide site variability based on the number of synonymous and nonsynonymous segregating sites expressed as percentage, respectively [60]; the average number of synonymous and nonsynonymous sites analyzed across loci were 155.6 and 582.4, respectively; *D*_S_ and *D*_A_, Tajima´s *D* [62] at synonymous and nonsynonymous sites, respectively; NA: not available; statistical significance of Tajima´s *D*:

*, *P* <0.05;

***, *P* <0.001.

The three genes displayed similar levels of synonymous variation in *N*. *apis* (pooled *π*_S_ values of 1.17%, 1.27% and 1.50%, for *PTP2*, *PTP3* and *RPB1* ^A^, respectively; these values were estimated by pooling all the sequences of each locus). The enlarged *RPB1* dataset (*RPB1* ^A+B^) produced comparable results (*π*_S_ = 1.68%) and was used hereafter. It is interesting to note that these pooled values are twice the observed average diversity across the seven samples (0.80%, 0.85% and 0.82% for *PTP2*, *PTP3* and *RPB1* ^A^, respectively). This discrepancy could reflect some level of differentiation among isolates and was further investigated in the “Population structure” section below.

The patterns of variation at these loci were also studied in *N*. *ceranae* (Table 2 and S2 Table). The pooled *π*_S_ values for *PTP2*, *PTP3* and *RPB1* were 1.00%, 0.85% and 1.58%, respectively and, in contrast to what was observed in *N*. *apis*, these estimates were very similar to the average diversities across samples (0.95%, 0.82% and 1.42%, for the same loci, respectively).

**Table 2:** Nucleotide diversity at three loci from *N*. *ceranae*: *PTP2*, *PTP3* and *RPB1*.

		*PTP2* ^a^							*PTP3* ^a^							*RPB1* ^b^						
Isolate	Origin	*N*	*π*_S_	*θ*_WS_	*D*_S_	*π*_A_	*θ*_WA_	*D*_A_	*N*	*π*_S_	*θ*_WS_	*D*_S_	*π*_A_	*θ*_WA_	*D*_A_	*N*	*π*_S_	*θ*_WS_	*D*_S_	*π*_A_	*θ*_WA_	*D*_A_
3	AUS															8	1.68	1.66	0.24	0.00	0.00	NA
4	AUS															8	0.81	1.23	-1.67*	0.13	0.19	-1.53
57	SPA	9	1.16	1.02	0.54	0.12	0.14	-0.58	8	1.10	1.08	-0.92	0.07	0.06	0.33	10	1.08	1.13	-0.24	0.05	0.09	-1.40
169	BRA	7	0.66	0.56	0.69	0.15	0.16	-0.27	9	1.14	1.07	0.24	0.22	0.28	-0.91	9	1.46	1.00	1.90	0.00	0.00	NA
253	SPA	10	1.53	1.47	0.11	0.12	0.21	-1.56	12	0.76	0.77	-0.06	0.29	0.46	-1.52	10	1.90	1.61	0.69	0.05	0.09	-1.40
376	CAN	8	0.60	0.53	0.41	0.08	0.07	0.33	6	0.70	0.76	-0.45	0.08	0.07	0.85	7	1.59	1.48	0.26	0.00	0.00	NA
377	CAN	6	1.11	1.21	-0.47	0.13	0.17	-1.13	8	0.88	1.34	-1.64	0.16	0.18	-0.30	10	1.87	1.61	0.41	0.05	0.09	-1.40
440	HUN	8	0.70	0.53	1.10	0.08	0.07	0.33	9	0.39	0.64	-1.51	0.13	0.17	-0.94	9	1.26	1.17	0.25	0.00	0.00	NA
526	NED	8	0.97	0.80	0.84	0.16	0.15	0.24	10	0.99	0.82	0.75	0.24	0.27	-0.38	7	1.85	1.48	1.18	0.07	0.10	-1.24
531	SLO	13	0.93	0.67	1.20	0.13	0.12	0.10	5	0.94	1.11	-1.09	0.21	0.22	-0.17	7	1.72	1.30	1.55	0.04	0.05	-1.01
839	ALG	7	0.40	0.56	-1.24	0.11	0.16	-1.24	10	0.35	0.62	-1.56	0.09	0.16	-1.56	7	0.00	0.00	NA	0.04	0.05	-1.01
911	TWN	6	0.74	0.60	1.03	0.30	0.34	-0.68	10	0.53	0.62	-0.51	0.14	0.16	-0.76	10	1.23	1.13	0.33	0.00	0.00	NA
912	SPA	8	0.87	1.33	-1.60	0.14	0.22	-1.45	12	0.70	0.96	-1.02	0.21	0.30	-1.17	6	0.61	0.80	-1.30	0.13	0.17	-1.23
1175	CRO	9	0.66	0.51	0.98	0.12	0.14	-0.58	11	0.57	0.79	-1.03	0.13	0.16	-0.51	7	1.99	1.86	0.27	0.04	0.05	-1.01
1244	ARG	7	1.06	0.85	1.11	0.09	0.08	0.56	11	0.40	0.59	-1.11	0.12	0.16	-0.75	10	1.59	1.45	0.36	0.05	0.09	-1.40
1251	HI	10	0.76	0.49	1.74	0.15	0.14	0.22	10	1.08	1.07	-0.54	0.30	0.43	-1.36	8	1.50	1.05	1.89	0.10	0.15	-1.45
1299	GRE	10	1.18	0.98	0.75	0.33	0.41	-0.84	12	1.06	0.96	0.33	0.11	0.10	0.22	8	0.97	1.23	-1.04	0.10	0.15	-1.45
1319	HI	7	1.06	0.85	1.11	0.09	0.08	0.56	5	1.17	1.39	-1.12	0.18	0.22	-1.05	11	1.55	1.09	1.62	0.05	0.09	-1.43
1324	HI	8	1.45	1.07	1.50	0.10	0.07	1.17	12	0.91	0.77	0.59	0.13	0.15	-0.38	6	1.51	1.20	1.39	0.13	0.17	-1.23
1610	USA	5	0.84	0.66	1.46	0.12	0.09	1.22	11	0.81	0.79	0.04	0.11	0.10	0.20	10	1.76	1.77	-0.10	0.05	0.09	-1.40
1994	CHI	11	1.09	1.41	-0.94	0.25	0.46	-1.90*	12	0.86	0.77	0.39	0.10	0.15	-1.38	10	1.69	1.29	1.28	0.05	0.09	-1.40
2032	SOL	12	1.20	0.92	1.03	0.14	0.13	0.15	11	1.11	1.78	-1.62	0.22	0.31	-1.22	10	1.39	1.12	0.90	0.10	0.18	-1.67
KI	JAP															8	1.60	1.23	1.35	0.00	0.00	NA
Pooled		169	1.00	2.19	-1.46	0.15	0.88	-2.34**	194	0.82	3.50	-2.21**	0.17	1.35	-2.49***	196	1.58	3.19	-1.54	0.05	0.78	-2.71***

^a^, Sequence data from [39];

^b^, sequence data for isolates 440, 1251 and 1324 from [32];

ALG: Algeria, ARG: Argentina, AUS: Australia, BRA: Brazil, CAN: Canada, CHI: Chile, CRO: Croatia, GRE: Greece, HI: Hawaii (USA), HUN: Hungary, JAP: Japan, NED: Netherlands, SLO: Slovenia, SOL: Solomon Islands, SPA: Spain, TWN: Taiwan, USA: United States of America; *N*: number of cloned sequences; *π*_S_ and *π*_A_, pairwise nucleotide diversity at synonymous and nonsynonymous sites expressed as percentage, respectively [59]; *θ*_WS_ and *θ*_WA_, nucleotide site variability based on the number of synonymous and nonsynonymous segregating sites expressed as percentage, respectively [60]; the average number of synonymous and nonsynonymous sites analyzed across loci were 178.3 and 650.7, respectively; *D*_S_ and *D*_A_, Tajima´s *D* [62] at synonymous and nonsynonymous sites, respectively; NA: not available; statistical significance of Tajima´s *D*:

*, *P* < 0.05;

**, *P* < 0.01;

***, *P* < 0.001.

In both species the levels of polymorphism at nonsynonymous positions were much lower than those observed at synonymous sites (Tables 1 and 2) and *θ*_W_ estimates were usually higher than those of *π* (resulting in pooled negative Tajima’s *D* values, especially in *N*. *ceranae*).

To verify if the observed patterns diversity and divergence at these three genes departed significantly from those of other genomic loci with available population data (*actin*, *Hsp70*, *HSWP4*, and *SWP30*) we performed a maximum likelihood HKA test [64]. *Actin* and *Hsp70* displayed lower diversity relative to their divergence levels (likelihood ratio test, *LRT* = 18.5, *P* < 0.001). No significant deviations were detected at any the other five loci. In addition, the ratio of nonsynonymous to synonymous divergence (*d*_N_/*d*_S_) was below unity for the seven loci, ranging from 0.02–0.04 for *actin*, *Hsp70* and *RPB1* to 0.21 for *SPW30* and *HSPW4* (S3 Table).

Considering the evidence for a recent population expansion in *N*. *ceranae* [32,38,39,42], we examined this possibility in *N*. *apis* by applying two alternative tests: Tajima’s *D* [62] and Fu’s *F*_*S*_ [63]. The former test can be used to compare the frequency spectrum of variants with neutral expectations, and revealed an excess of low frequency synonymous variants in *N*. *apis*. Although this deviation was not significant at individual loci (Table 1), it was significant when data from *PTP2*, *PTP3* and *RPB1* were combined (*D*_*S*_ = -1.82, *P* = 0.034). Similar results were obtained in *N*. *ceranae*, where the combined data revealed a significant excess of low frequency synonymous variants (*D*_*S*_ = -2.93, *P* < 0.002). The *F*_*S*_ test [63] provided additional evidence for a significant excess of haplotypes in *N*. *apis PTP2* and *RPB1* genes, as compared with neutral expectations (Table 3). *N*. *ceranae* sequences obtained from *A*. *mellifera* displayed a similar pattern (Table 3).

**Table 3:** *F*_S_ test for detecting population expansion.

	*Fs*					
Dataset	*PTP2*		*PTP3*		*RPB1*	
*N*. *apis*	-18.66	***	-8.17	ns	-87.22	***
Lineage A	-1.52	ns	0.46	ns	-3.54	*
Lineage C	-13.24	***	-5.70	**	-34.15	***
Lineage M					-0.77	ns
*N*. *ceranae* ^a^ ^,^ ^b^	-34.35	***	-120.89	***	-77.55	***

*Fs*: Fu's *Fs* [63]; The significance was evaluated by comparing the values of the statistic, and the observed levels of recombination per gene, with a null distribution generated by 1,000 coalescent simulations (ns, non-significant; *, *P* < 0.05; **, *P* < 0.01; ***, *P* < 0.001);

^a^, sequence data for *PTP2* and *PTP3* from [39];

^b^, sequence data for isolates 440, 1251 and 1324 (*RPB1*) from [32];

the remaining ones are from this work. Lineages A, C and M indicate the evolutionary lineage of the honey bee colonies where the isolates come from (isolate 410 was excluded from this analysis).

Given the possibility that the *N*. *apis* population was subdivided in two demes (see the “Population structure” section below), one in lineage A honey bees (from Africa and the Iberian Peninsula) and another one in the European honey bee lineages C and M, the *D* and *F*_S_ statistics were estimated separately in both groups. Tajima’s *D* for lineage C isolates was significant both at synonymous and nonsynonymous sites (-2.18, *P* < 0.015 and -3.30, *P* < 0.0001, respectively), but no significant skew was observed at synonymous sites among isolates collected from honey bees of lineages A or M (*D*_S_ = -0.19 and 1.83, ns; respectively.). The *F*_S_ test produced a similar scenario (Table 3).

### Recombination

The estimate of the population scaled recombination rate (*ρ*) was significantly greater that zero at *PTP2* and *RPB1* in *N*. *apis* (Table 4) and several recombination events were detected at these two loci (*Rm* = 2 and 6, *Rh* = 2 and 14, respectively).

**Table 4:** Statistics used to detect recombination in *N*. *apis*.

Dataset	Locus	*N*	*Rm*	*Rh*	*ρ*	*P*
*N*. *apis*	*PTP2*	63	2	2	36	*
	*PTP3*	50	0	0	2	ns
	*RPB1*	208	6	14	11	***
*N*. *ceranae*	*PTP2* ^a^	169	5	10	68	ns
	*PTP3* ^a^	194	3	6	61	*
	*RPB1* ^b^	196	6	12	14	*

*N*, number of cloned sequences; *Rm*, minimum number of recombination events [69]; *Rh*: lower bound on the number of recombination events [70] (http://www.stats.ox.ac.uk/~myers/RecMin.html); *ρ*, estimate of the population scaled recombination rate; *P*, probability of Lkmax ≤ estimated in a likelihood permutation-based test as implemented in LDhat [72]; ns, non-significant,

*, *P* < 0.05 and ***, *P* < 0.001;

^a^, sequence data from [39];

^b^, sequence data for isolates 440, 1251 and 1324 from [32];

the remaining ones are from this work.

The outcome of these tests did not change after removing singleton mutations, which usually contain little information and could cause interferences in the assessment of recombination. In addition, the four-allele combinations expected after a recombination event were found both in individual samples (e.g. AT, TT, AC and TC at positions 276 and 453 of *PTP2* in sample 854 –haplotypes 1, 15, 23 and 24, respectively–; S4 Table) and in samples from different populations (e.g. CC, CT, TC and TT at positions 771 and 963 of *RPB1* ^A+B^; S7 Table), further supporting the existence of low, although statistically significant, levels of recombination in *N*. *apis*. Likewise, evidence for recombination was found in *N*. *ceranae*, although in this case it only reached statistical significance for *PTP3* and *RPB1* (Table 4).

### Sex and meiotic loci

A Blastp analysis of the genomes of *N*. *apis* and *N*. *ceranae* revealed the presence of several components of a sex-related locus [78] that encode a *triose phosphate transporter* (*TPT*), a *high-mobility group* (*HMG*), and an *RNA helicase* (with accession numbers EQB61312, EQB61310, EQB60627 and KKO76186, KKO76188, KKO75161, respectively). In both species *TPT* and *HMG* were syntenic and harbored an additional predicted ORF between them, whereas the *RNA helicase* was unlinked to the former.

These genomes also contained meiotic genes, although not all of them presented orthologs in both species (S8 Table). However, it must be noted that these results should be taken with caution due to the difficulty of distinguishing orthologs encoding different members of gene families (i.e. *Smc1* and *Smc4* in *N*. *ceranae*; S8 Table).

### Population structure

**Fig 1:**
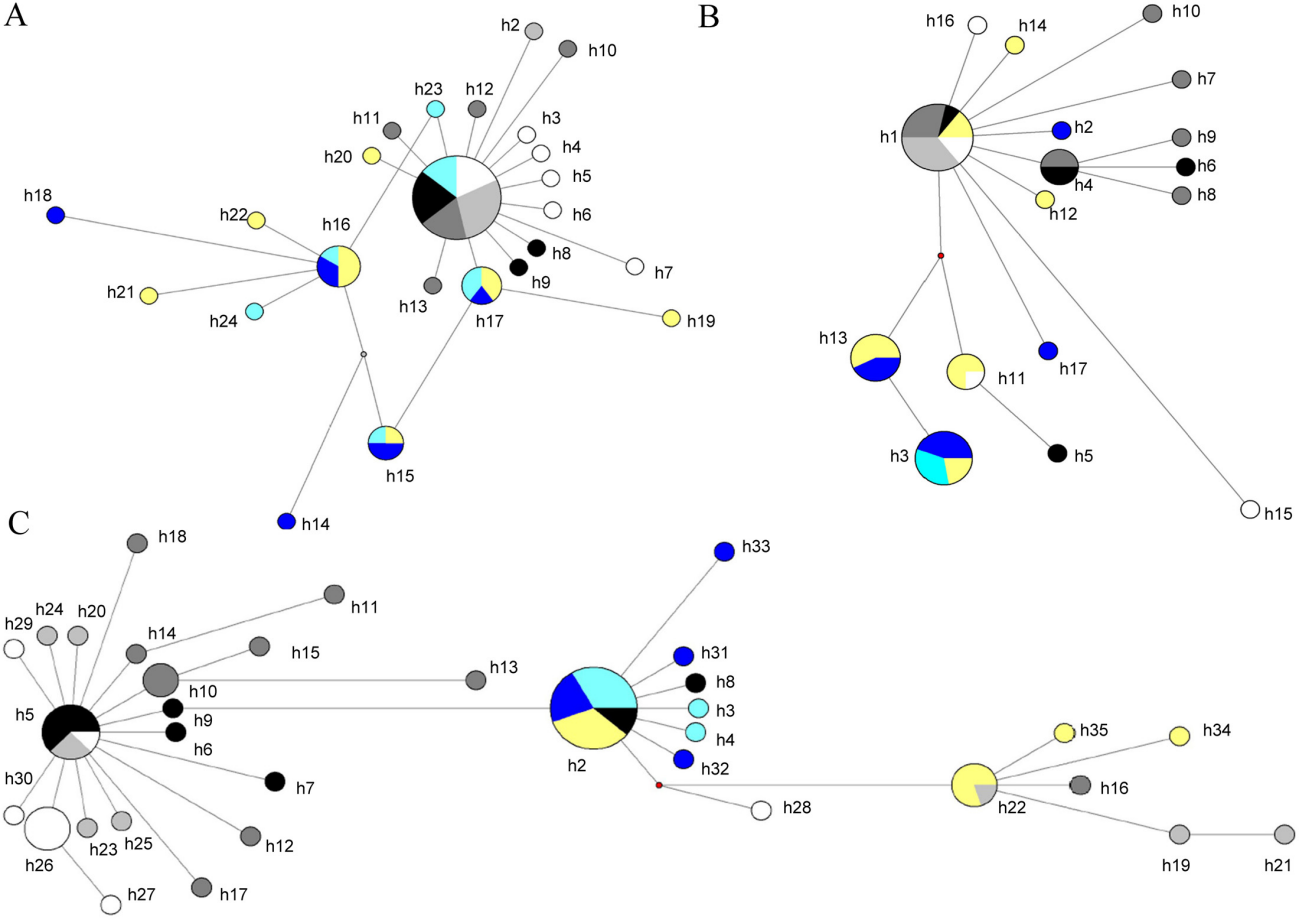
Median-joining haplotype network for three *N*. *apis* loci according to their geographical origin and *A*. *mellifera* lineage: *PTP2* (A), *PTP3* (B) and *RPB1* (C). Haplotypes are depicted by circles, the width being proportional to their frequencies. Color codes are as follows; blue: lineage A (light blue: isolate 854 (Algeria); dark blue: isolate 399 (Spain)); yellow: lineages A/M (isolate 410 (Spain)); greyscale: lineage C (black: isolate 204 (Argentina); dark grey: isolate 381 (Canada); light grey: isolate 52 (Slovenia); white: isolate 174 (Turkey)); red dots represent median vectors (hypothesized haplotypes required to connect existing sequences within the network with maximum parsimony).

*N*. *apis* exhibited high levels of haplotype diversity at the three loci under study (*Hd* = 0.79–0.91). To explore the distribution of the haplotypes among samples these were plotted into networks (Fig 1), revealing the following patterns: (i) all loci presented a reduced number of common haplotypes (e.g. h1 in *PTP2* and in *PTP3*, and h2 and h5 in *RPB1*); (ii) most other haplotypes differed from these by a reduced number of substitutions (usually one or two); and (iii), there was a hinted association among haplotypes obtained from Spanish and Algerian samples. For example, *PTP2* haplotypes h15, h16, h17 were shared by the three samples from these two countries (Fig 1A), and most other closely linked haplotypes (e.g. h14, h18, h19, h21, h22 and h24) were found in either of them. A similar effect was observed for *PTP3* haplotypes h3 and h13 (Fig 1B), and h2 of *RPB1* (Fig 1C). This raised the possibility of a structuring of the parasite and host populations, which was not apparent in *N*. *ceranae* networks (S1 Fig), and it was further explored by determining the evolutionary lineage of all sampled honey bee colonies and by quantifying the relative contribution of three covariance components to the observed parasite haplotype diversity: (i) covariance within isolates (i.e., within honey bee colonies), (ii) among isolates obtained from honey bees of the same evolutionary lineage, and (iii) among host evolutionary lineages. Most *N*. *apis* isolates were collected from *A*. *mellifera* colonies of lineage C, except isolates 399 and 854 (from Spain and Algeria) which were of lineage A, and isolates 382 (Canada) and 411 (Chile) which belonged to lineage M (S1 Table). A third isolate from Spain, 410, displayed mixed results, with evidence for the presence of honey bees of both lineages A and M, probably due to drifting workers and the existence of colonies of both evolutionary lineages in the same apiary (S1 Table).

The analysis of molecular variance revealed a structured *N*. *apis* population, where between 20 and 34% of the total variance at the three loci corresponded to differences among host-lineages (*P* < 0.05, in permutation tests; Table 5). These results held irrespective of whether sample 410 was considered as belonging to lineage A or M. Differences among haplotypes of the same isolate accounted for the best part of the variance (between 62 and 70%), and differences among isolates of the same lineage represented ≤11% of the variance. In *N*. *ceranae* the differentiation of haplotypes within isolates was even greater (between 91 and 99%) but, contrastingly, the differences among host-lineages were not significant as they represented just a tiny fraction of the observed variance (< 4%, Table 5).

**Table 5:** Analysis of molecular variance (AMOVA) in *N*. *apis* haplotypes according to *A*. *mellifera* lineages.

Dataset	Locus	Source of variation	d.f.	*SS*	*VC*	% *var*	*P*
*N*. *apis*	*PTP2*	Among lineages	1	17.2	0.51	32.6	*
		Among isolates within lineages	5	6.1	0.02	1.3	ns
		Within isolates	56	58.7	1.05	66.1	***
	*PTP3*	Among lineages	1	13.9	0.50	33.8	*
		Among isolates within lineages	5	6.8	0.07	4.6	*
		Within isolates	43	38.9	0.90	61.6	***
	*RPB1*	Among lineages	2	54.3	0.56	19.6	**
		Among isolates within lineages	19	94.1	0.32	10.8	***
		Within isolates	186	374.3	2.01	69.6	***
*N*. *ceranae*	*PTP2* ^a^	Among lineages	2	6.0	0.04	3.5	*
		Among isolates within lineages	17	20.5	0.01	0.9	ns
		Within isolates	149	166.3	1.12	95.6	ns
	*PTP3* ^a^	Among lineages	2	242.1	-13.96	-2.2	ns
		Among isolates within lineages	17	14014.6	20.29	3.2	ns
		Within isolates	174	109558.1	629.64	99.0	ns
	*RPB1* ^b^	Among lineages	2	10.0	0.04	1.9	ns
		Among isolates within lineages	20	63.9	0.14	6.7	*
		Within isolates	173	339.7	1.96	91.4	**

^a^, Sequence data from [39];

^b^, sequence data for isolates 440, 1251 and 1324 from [32];

the remaining ones are from this work; isolate 410 was considered as if sampled from a lineage A honey bee colony (see text); d.f., degrees of freedom; *SS*, sum of squares; *VC*, variance components; % var, percentage of variation; *p*, probability of a random variance component value ≤ observed value, in 3024 permutations;

*, *P* < 0.05; **^,^ *P* < 0.01 and ***, *P* < 0.001; ns, non-significant.

A pairwise analysis of *N*. *apis* differentiation between host lineages uncovered that most variation occurred between lineage A and the other two lineages, which were otherwise indistinguishable (Table 6). *K*_ST_*, which measures the average pairwise differences within populations with respect to the total, revealed significant differentiation between *N*. *apis* sequences obtained from *A*. *mellifera* colonies of lineages A and C (*K*_ST_* between 0.09 and 0.25, *P* < 0.001, Table 6) or between A and M (*K*_ST_* = 0.30, *P* < 0.001). Similarly, *S*_nn_, which estimates how often related sequences are found in the same population, reached significant values between group A and C (*S*_nn_ = 0.82–0.93, *P* < 0.001) and between A and M (*S*_nn_ = 1.00, *P* < 0.001). Both tests failed to detect significant differences between sequences obtained from lineages C and M.

**Table 6:** Analysis of population differentiation in *N*. *apis* according to *A*. *mellifera* lineages.

Dataset	Locus	Host Lineages	*K*_ST_*	*P*	*S*_nn_	*P*
*N*. *apis*	*PTP2*	A & C	0.16	***	0.82	***
	*PTP3*	A & C	0.25	***	0.95	***
	*RPB1*	A & C	0.09	***	0.93	***
		A & M	0.30	***	1.00	***
		C & M	0.00	ns	0.81	ns
*N*. *ceranae*	*PTP2* ^a^	A & C	0,02	**	0,69	*
		A & M	0,02	*	0,55	ns
		C & M	0,00	ns	0,69	ns
	*PTP3* ^a^	A & C	0,00	ns	0,67	*
		A & M	0,01	*	0,61	**
		C & M	0,00	ns	0,73	**
	*RPB1* ^b^	A & C	0.02	*	0.68	ns
		A & M	0.00	ns	0.54	ns
		C & M	0.00	ns	0.76	ns

^a^, sequence data from [39];

^b^, sequence data for isolates 440, 1251 and 1324 from [32];

the remaining ones are from this work; isolate 410 was excluded; *K*_ST_* [73], estimates the amount of within-deme nucleotide diversity relative to the overall diversity; *S*_nn_ [74], measures how often related sequences are found in the same deme; *P*, significance in permutation tests: ns, non-significant;

*, *P* < 0.05; **, *P* < 0.01; ***, *P* < 0.001.

The divergence between two populations is a direct function of the mutation rate times twice the number of generations since their split. Considering that: (i) the net divergence (*D*a) between *N*. *apis* from lineage A and C/M colonies is 0.002. (ii) The number of spores can double as fast as every 24 hours (e.g. [79,80]). (iii) The substitution rate in these pathogens is about two times faster than that observed in other fungi (as estimated for *Encephalitozoon cuniculi* [81]), and that (iv) the per site mutation rates in fungi is of the order of 7.2 x 10^−11^ for *Neurospora crassa* or 2.2 x 10^−10^ for *Saccharomyces cerevisiae* [82], the split between the two parasite populations can be dated between 6,200 and 19,000 years ago.

## Discussion

Here we report a population genetic analysis of *N*. *apis* based on the study of the patterns of diversity of three unlinked single copy genes: *PTP2* and *PTP3*, which encode polar tube proteins (reviewed in [83]), and the largest subunit of the RNA polymerase II (*RPB1*), a housekeeping gene that has been frequently used as phylogenetic marker in microsporidian species [84,85]. The levels of synonymous variation at these unlinked coding genes should be a good proxy for the extant neutral variation of the species [86]. The fact that they are single copy markers makes them a preferred choice than the commonly used ribosomal loci, as substantial levels of genetic variation and recombination between paralogous rDNA copies have been previously reported in microsporidia [36]. So far only Ironside [44] has published diversity data for a single copy locus (*RPB1*) in *N*. *apis*.

The three loci analyzed displayed similar average levels of synonymous diversity (*π*_S_), about 1% (Table 1), analogous to what was found for these and other loci in *N*. *ceranae* [32,38,39,41], and somewhat higher than those estimated for *RPB1* (0.41%) in cloned sequences from a single isolate of *N*. *apis* [44]. In terms of diversity at all sites *N*. *ceranae* and *N*. *apis* displayed values of the order of 0.40%, which are lower than those described in *N*. *bombycis* (1.83%, [44]), but higher than those of other microsporidia of the genus *Hamiltosporidium* (between 0.06% and 0.28%, [87]).

Although it has been postulated that the polar tube proteins could be factors of virulence and thus subject to adaptive selection [88], the MLHKA test revealed that the relative levels of diversity and divergence of the *PTP* loci do not differ from those observed at three other unlinked loci (including a housekeeping gene, *RPB1*), which suggests that they evolve under the effect of similar evolutionary forces. Consistently low *d*_N_/*d*_S_ values can be reconciled with a predominant effect of purifying selection over the seven loci. Although the large synonymous divergence between these species means that these results should be taken with caution, the fact that it applies to all loci supports that the genes used in the current study are a good proxy of the patterns of variation across the parasites’ genome.

The detection of substantially lower variation (*π*_A_) coupled with significantly negative Tajima’s *D*_A_ values at nonsynonymous sites at the three loci in both species indicate that amino acid replacement variants are readily removed from the populations, which reflects that these loci are likely to be functional and subject to purifying selection, as previously suggested in *N*. *apis* [44] and *N*. *ceranae* [32,36,38,39,44] for these and other genes. This fits well with the finding of just 29 putative pseudogenes in the genome of *N*. *ceranae* [42], which indicates that the majority of coding sequences retained in these reduced genomes [33,48] are essential for the survival of these parasites. The relatively lower variability at a*ctin* and *Hsp70* can probably be attributed to the effect of negative selection at linked deleterious sites (background selection) at these loci [89], which is consistent with the strong purifying selection—low *d*_N_/*d*_S_ values—observed in these highly conserved genes [90,91] and the low recombination rates reported for these parasites [40] (see below).

The results of the Tajima´s *D* test at silent sites revealed an overall excess of low frequency variants in the two parasite species (Tables 1 and 2). Although some of these could correspond to nucleotide misincorporations introduced during the PCR process (despite the use of a high-fidelity enzyme blend), previous assays using either invariant DNA templates [38,39] or single DNA molecules [40], confirmed that the vast majority of the mutations detected in *N*. *ceranae* were actually present in the sample mixture, and that no error-prone bias was brought throughout the procedure. The same pattern was also observed at the genomic level [42], so there are no reasons to think that this would be different in *N*. *apis*.

All isolates presented substantial levels of nucleotide diversity (Tables 1 and 2). In fact, many of them harbored various distinct haplotypes, sometimes as many as nine (e.g. S7 Table). Given that the three genes are present as a single copy in the genome of both parasites, there are two possible and non-mutually exclusive explanations for the observed within-isolate variation: one is to assume that the two nuclei present in each cell are diploid, as it has been recently proposed for *N*. *ceranae* [42], so that they can harbor up to four different haplotypes for each loci, and the other is the existence of genetic heterogeneity among parasites in each host colony (mixed infections) [32,39,41].

At any rate, the accumulation of alleles at low frequencies observed in these two species is compatible with a demographic growth, in which most mutations present in the expanding populations have a recent origin and, therefore, are rare [92]. The greater *D*_*S*_ value obtained for *N*. *ceranae* in the combined sample (*D*_*S*_ = -2.93, *P* < 0.002), suggests that the expansion of this population might have taken place more recently or it has been more accentuated than that experienced by *N*. *apis* (*D*_*S*_ = -1.82, *P* < 0.034). This would agree with its recent jump to *A*. *mellifera* and spread throughout the worldwide distribution range of its new host [32,38,39,41,42].

*A*. *mellifera* originated between 6 and 8 million years ago somewhere in Asia, where all other species of the genus are confined, and from where it expanded to its historical geographic distribution range across sub-Saharan Africa, Europe and Western Asia [93,94]. The species now comprises several locally adapted and anatomically distinct subspecies, which split between 0.3 and one million years ago and can be clustered into four major groups: lineage A, includes subspecies that can be found in Africa and the Iberian Peninsula; lineage M is distributed along Western and Northern Europe; lineage C, in South Eastern Europe, and lineage O, in the Middle East and Western Asia [93,94]. Our results suggest that between 20% and 34% of the genetic variance of the *N*. *apis* population corresponds to differences between samples collected from honey bee colonies of different lineages (Table 5). It should be noted that the sampling scheme might influence the observed frequency spectra of variants, as the retrieval of alleles from distant locations of a structured population is likely to cause departures from neutral expectations assuming panmixia. The reduced between-sample variation in *N*. *ceranae* means that this effect is unlikely in this species, but the evidence for genetic differentiation between *N*. *apis* collected from different host lineages (lineage A *vs*. C or M) suggests that the assumed panmixia might not be met. The separate analysis of the parasite subpopulations revealed that only those isolated from European honey bees of lineage C depart from mutation-drift equilibrium.

Remarkably, the observed structure of the parasite population does not match that of the host: lineages C and M display the highest differentiation amongst the four *A*. *mellifera* lineages [93,94]. In contrast, *N*. *apis* isolates from these lineages are genetically indistinguishable. This lack of a full correspondence between the structures of parasite and host populations suggests that they only share a fraction of their demographic history. Indeed, the split of the *N*. *apis* populations retrieved from honey bee lineages A and C (or M) was dated between 6,200 and 19,000 years ago, that is just after the Last Glacial Maximum, about 20,000 years ago [93]. Thus, a reduction of the parasite’s geographic distribution range during the last glacial period might have prompted the isolation of the two populations. Contrastingly, the absence of genetic differentiation between the parasites from lineages C and M suggests that this population has spread across Europe recently (which is also consistent with the results of the Tajima´s *D* and *Fs* tests), and much later than the honey bees did. This expansion might have been associated with the practice of beekeeping by humans, whose origin has been traced to the Middle East and Egypt about five thousand years ago [95], and also with the human-driven colonization by *A*. *mellifera* of the New World, East Asia and Oceania in the last few centuries [94,96].

Evidence for low levels of recombination from nucleotide variation data had been previously reported [32,36,38,39,41,42], and further supported by Single Genome Amplification (SGA) analysis, in *N*. *ceranae* [40]. This new evidence for a second *Nosema* species, suggests that recombination might be a common feature of the genus. Genetic exchange between chromosomes is crucial in the evolution of organisms, and its detection has important consequences since it can generate new genetic combinations that result in individuals better adapted to confront novel environments or hosts.

If exclusively clonal reproduction is assumed, high levels of genetic differentiation would be expected between homologous sequences in the two nuclei of each individual (an adaptation of the Meselson effect for a diplokaryon), as observed in the asexual microsporidian *Hamiltosporidium tvarminnensis* [87]. But the absence of genetic structuration of the haplotypes retrieved in each colony, along with the observed neutral diversity within samples and the evidence for genetic recombination, suggest that there might exist mechanisms for occasional genetic flow between the nuclei. Whether this exchange takes place between nuclei of the same cell or between those of different cells during the multinucleated stages of the merogonia [97] remains to be determined.

Genetic exchange can occur during meiosis in sexual stages of the life cycle but also during parasexual processes such as mitotic crossover, non-homologous recombination and gene conversion. Although both mechanisms have been proposed in microsporidians (e.g.*Encephalitozoon cuniculi* [98], *Hamiltosporidium magnivora* [87], *Nematocida* [99] or *Nosema* spp. [36,44,100]), their occurrence and frequency are still a matter of debate as, in absence of cytological observation, the outcomes of these processes are difficult to distinguish [101,102]. The finding of a putative sex-related locus in *N*. *ceranae* and in *N*. *apis* goes in line with similar evidence in other microsporidians [76,87,99,103], but it should not be taken as a definite proof of sexual reproduction, which would require the presence of idiomorphs of this locus in different isolates and their expression during the mating phase [76]. The same could be said regarding the existence of core meiotic genes in these genomes [76,99,103,104] whose detection, although suggestive that meiosis may occur, does not ensure their functionality during this process [103].

The genetic diversity patterns at the three loci analyzed in this study suggest that *N*. *apis* and *N*. *ceranae* have experienced different recent evolutionary histories and provide new data on the relationship between these parasites and the honey bees that host them. In addition, they extend the evidence for genetic recombination to a second species of the genus, further supporting the idea that mechanisms of genetic exchange between chromosomes play an important role in modeling the genetic configuration of these organisms. However, further studies are needed in order to determine the extent to which the observed patterns extend to other parts of these species’ genomes, to elucidate the molecular mechanisms responsible for the observed recombination and whether or not it implies sexual reproduction.

## Supporting Information

S1 Fig Median-joining haplotype network for three *N*. *ceranae* loci according to their *A*. *mellifera* lineage: *PTP2* (A), *PTP3* (B) and *RPB1* (C). Haplotypes are depicted by circles, the width being proportional to their frequencies (only shared haplotypes are named). Color codes are as follows; blue: lineage A (isolates 839 (Algeria), 57 and 253 (Spain), 169 (Brazil)); yellow: lineage M (isolates 912 (Spain), 526 (Netherlands), 1251 (Hawaii)); grey: lineage C (isolates 1244 (Argentina), 3 and 4 (Australia), 376 and 377 (Canada), 440 (Hungary), 531 (Slovenia), 911 (Taiwan), 1175 (Croatia), 1299 (Greece), 1319 and 1324 (Hawaii), 1610 (USA), 2032 (Solomon Islands), 1994 (Chile), KI (Japan)); red dots represent median vectors (hypothesized haplotypes required to connect existing sequences within the network with maximum parsimony).
(TIF)

S1 Table Origin and accession numbers of *N*. *apis* sequences obtained from *A*. *mellifera* honey bees. (XLS)

S2 Table Origin and accession numbers of *N*. *ceranae* sequences obtained from *A*. *mellifera* honey bees. (XLS)

S3 Table Ratio of nonsynonymous to synonymous divergence (*d*_N_/*d*_S_) between *N*. *apis* and *N*. *ceranae* (Yang and Nielsen method). (XLS)

S4 Table Number of occurrences and nucleotide variants of *PTP2* ^A^ haplotypes from *N*. *apis*. (XLS)

S5 Table Number of occurrences and nucleotide variants of *PTP3* ^A^ haplotypes from *N*. *apis*. (XLS)

S6 Table Number of occurrences and nucleotide variants of *RPB1* ^A^ haplotypes from *N*. *apis*. (XLS)

S7 Table Number of occurrences and nucleotide variants of *RPB1* ^A+B^ haplotypes from *N*. *apis*. (XLS)

S8 Table Meiotic genes in different microsporidian species. (XLS)
