# Supplementary figures and images for: Population Genetics of Nosema apis and Nosema ceranae: One Host (Apis mellifera) and Two Different Histories
Source: PLoS One. 2015 Dec 31;10(12):e0145609. doi: 10.1371/journal.pone.0145609 (PMC4699903; doi:10.1371/journal.pone.0145609)

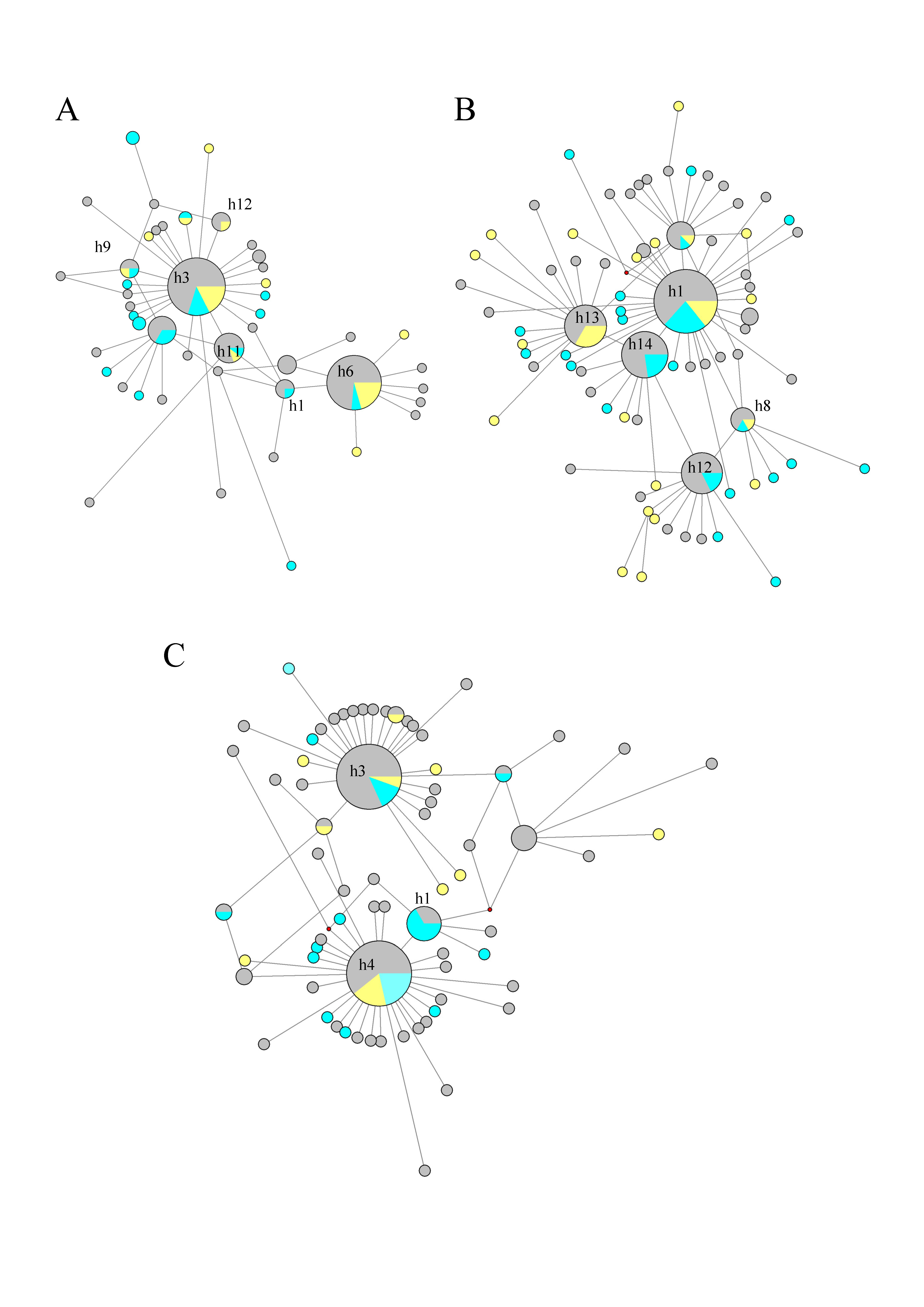

Supplement: S1 Fig — Haplotypes are depicted by circles, the width being proportional to their frequencies (only shared haplotypes are named). Color codes are as follows; blue: lineage A (isolates 839 (Algeria), 57 and 253 (Spain), 169 (Brazil)); yellow: lineage M (isolates 912 (Spain), 526 (Netherlands), 1251 (Hawaii)); grey: lineage C (isolates 1244 (Argentina), 3 and 4 (Australia), 376 and 377 (Canada), 440 (Hungary), 531 (Slovenia), 911 (Taiwan), 1175 (Croatia), 1299 (Greece), 1319 and 1324 (Hawaii), 1610 (USA), 2032 (Solomon Islands), 1994 (Chile), KI (Japan)); red dots represent median vectors (hypothesized haplotypes required to connect existing sequences within the network with maximum parsimony). (TIF) [file pone.0145609.s001.tif]
